# Imiquimod Does not Affect Shedding of Viable Chlamydiae in a Murine Model of *Chlamydia trachomatis* Genital Tract Infection

**DOI:** 10.1080/10647440300025503

**Published:** 2003

**Authors:** Kyle H. Ramsey, Namir Shaba, Kevin P. Cohoon, Kevin A. Ault

**Affiliations:** 1 Department of Microbiology Chicago College of Osteopathic Medicine Midwestern University 555 31st Street, Downers Grove Downers Grove IL 60515 USA; 2 Department of Obstetrics and Gynecology University of Iowa College of Medicine Iowa City IA USA

## Abstract

*Objective:* We postulated that either oral or vaginal administration of the immune response modifier imiquimod would decrease vaginal shedding of *Chlamydia trachomatis*, mouse pneumonitis strain (MoPn), in a murine model.

*Methods:*  Female BALB/c mice were infected intravaginally with*C. trachomatis*  (MoPn) and were administered imiquimod either orally (30 mg/kg) or vaginally (10 μl of 5%imiquimod cream) prior to infection and every second day after infection for a total of four doses. The course of infection was monitored by collecting cervical–vaginal swabs and isolation in HeLa 229 cell culture. To determine whether the drug affected T helper type 1 or T helper type 2 immune response polarization, immunoglobulinG(IgG) subclass antibody responses were assessed at day 56 after infection.

*Results:* There was no significant difference in the course of infection when imiquimod-treated mice were compared with sham-treated controls, regardless of whether the drug was administered orally or vaginally. IgG subclass antibody responses, and by extension, T helper type 1 to T helper type 2 immune response polarization, were also unaffected.

*Conclusions:* Imiquimod has no efficacy in controlling*C. trachomatis*  (MoPn) infection in the murine model.


Infect Dis Obstet Gynecol 2003;11:81–87

### Imiquimod does not affect shedding of viable chlamydiae in a murine model of *Chlamydia trachomatis* genital tract infection

**Kyle H. Ramsey[1], Namir Shaba[1], Kevin P. Cohoon[1] and Kevin A. Ault[2]**

[1]*Department of Microbiology, Chicago College of Osteopathic Medicine, Midwestern University, Downers Grove, IL, and*
[2]*Department of Obstetrics and Gynecology, University of Iowa College of Medicine, Iowa City, IA*

***Objective:*** We postulated that either oral or vaginal administration of the immune response modifier imiquimod would decrease vaginal shedding of *Chlamydia trachomatis*, mouse pneumonitis strain (MoPn), in a murine model.
***Methods:*** Female BALB/c mice were infected intravaginally with *C. trachomatis* (MoPn) and were administered imiquimod either orally (30 mg/kg) or vaginally (10 μl of 5% imiquimod cream) prior to infection and every second day after infection for a total of four doses. The course of infection was monitored by collecting cervical–vaginal swabs and isolation in HeLa 229 cell culture. To determine whether the drug affected T helper type 1 or T helper type 2 immune response polarization, immunoglobulin G (IgG) subclass antibody responses were assessed at day 56 after infection.
***Results:*** There was no significant difference in the course of infection when imiquimod-treated mice were compared with sham-treated controls, regardless of whether the drug was administered orally or vaginally. IgG subclass antibody responses, and by extension, T helper type 1 to T helper type 2 immune response polarization, were also unaffected.
***Conclusions:*** Imiquimod has no efficacy in controlling *C. trachomatis* (MoPn) infection in the murine model.

Key words: CHLAMYDIA TRACHOMATIS; IMIQUIMOD; MOUSE; IMMUNITY; TREATMENT

Imiquimod (Aldara, R-837, S-26308), an imidazoquinolinamine, is a proven immune response modifier that induces endogenous cytokine secretion from monocytes or macrophages (e.g. interferon-α, interferon-γ, tumor necrosis factor-α, interleukin (IL)-1β, and IL-6, IL-8 and IL-12), and similarly elicits interferon-α and IL-6 from epithelial cells[1–3]. It also enhances antigen presentation by dendritic cells and the expression of the inducible form of nitric oxide synthase. Thus the cytokine milieu that is generated by imiquimod is thought to lead to type 1 T helper cell (Th1) dominance and enhanced cell-mediated immunity[3]. In mice, cell-mediated immunity and Th1 cytokine polarization of an immune response are known to influence the isotype switch of B-cells toward the production of immunoglobulin G2a (IgG2a) antibodies such that a surrogate marker for a Th1 polarized immune response is predominance of IgG2a production[4].

Clinically, imiquimod is approved for the topical treatment of genital warts caused by human papilloma virus (HPV) infections. However, it also has demonstrated efficacy in humans and in animal models for the treatment of non-genital warts and mollusca[5,6], herpes simplex virus (HSV)[7–11], cytomegalovirus[12] and cancerous lesions, including initial squamous-cell and

Correspondence to: Kyle H. Ramsey, PhD, Department of Microbiology, Chicago College of Osteopathic Medicine, Midwestern University, 555 31st Street, Downers Grove, IL 60515, USA. Email: kramse@midwestern.edu

© 2003 The Parthenon Publishing Group basal-cell carcinoma in immunocompetent and immunosuppressed patients[2]. Recent studies have also shown efficacy for imiquimod in the treatment of experimental murine and drug-resistant human cutaneous leishmaniasis[13,14]. In each instance, imiquimod is thought to exert its effect through the induction of interferons and certain other cytokines that enhance Th1 responses[15].

Immunity to chlamydial infections in humans is not well defined, but probably involves a contribution of both local antibody production and T-cell responses[16]. *In vitro*, the effect of interferon-γ on the restriction of intracellular replication of *Chlamydia* in human epithelial cells is particularly well characterized[17]. Although they have not yet been fully defined, protective immune mechanisms have been better characterized in murine models where a Th1 polarized response is of central importance[18]. Briefly, resolution of *C. trachomatis* infection in the mouse is known to require major histocompatibility complex (MHC) class II antigen processing by dendritic cells and the subsequent activation of T-cells that produce interferon-γ to migrate to the site of infection[19–26]. A similar dependence on T-cells is required for the prevention of subsequent infections, but a role for B-cell-mediated antigen processing in eliciting this response appears to be crucial[27,28].

Given the importance of a cell-mediated immune response in resolving chlamydial infections, we postulated that chlamydial infection would be decreased in magnitude or over time by the administration of imiquimod in a murine model, and that this would be accomplished by enhancing a cytokine profile characteristic of that observed in Th1 polarized immune responses. Alternatively, imiquimod could enhance the local cytokine milieu (e.g. interferons) at the site of infection, which would either directly lead to decreased shedding of *C. trachomatis* or enhance Th1 polarization at the onset of the response.

### MATERIALS AND METHODS

### Infection, infection assessment and experimental design

Six to eight-week-old BALB/c mice were purchased from Harlan-Sprague-Dawley (Indianapolis, IN) and allowed to acclimatize for one week. Mice were allowed access to food and water *ad libitum*. Seven days prior to infection, mice were pretreated with 2.5 mg of medroxyprogesterone acetate (DepoProvera, Upjohn, Kalamazoo, MI) and inoculated intravaginally with 200 $ID_{50}$ ($10^4$ inclusion-forming units, IFUs) of *C. trachomatis*, mouse pneumonitis strain (MoPn), exactly as described by Cotter and co-workers[26,29]. Infection was assessed by collecting cervical–vaginal swabs 4, 7, 10 and 14 days after infection and weekly thereafter. Culture of swab-collected material in HeLa 229 cell monolayers in 24 well plates was performed, and IFUs were counted by indirect fluorescent antibody staining and viewing on an inverted fluorescent microscope[26]. In the first experiment, mice were administered imiquimod (30 mg/kg suspended in 0.25 ml of sterile saline) by gavage 3 days prior to infection and again on the day of infection and at 3, 6 and 9 days after infection. Control mice were administered a similar volume of saline alone, also by gavage.

In a second experiment, 10 μl of 5% imiquimod cream were applied intravaginally using a blunted small-bore (26-gauge) 5/8th-inch straight gavage needle on a 0.5-ml syringe at 2 days prior to infection and again 1, 3 and 6 days after infection. Cervical–vaginal swabs were collected at identical time points to those in the first experiment, thereby avoiding the collection of swabs and administration of the drug on the same days, which could possibly interfere with the sampling method or with the effect of the drug.

### IgG subclass antibody response determination

In order to determine whether the administration of imiquimod enhanced Th1 responses or induced a shift in Th1 to Th2 immune response polarization, IgG subclass antibody responses were assessed using an enzyme-linked immunosorbent assay (ELISA). Briefly, on the day of infection and 56 days after infection, mice were anesthetized with pentobarbital and blood was collected via puncture of the retro-orbital venous plexus. Plasma IgG antibody subclass responses were assessed on serial two-fold dilutions (beginning with a 1:10 dilution) using a previously published method[30].

We modified this method to use secondary phosphatase-labeled rat antibody against mouse IgG1, IgG2a, IgG2b or IgG3 (Southern Biotechnology Associates, Birmingham, AL). Optical densities were read using a Dynatek® MR5000 automated 96-well spectrophotometer with Biolinks® software. After background subtraction (of wells receiving secondary antibody only), an arbitrary cut-off value at an $OD_{405}$ of 0.1 was considered to be a positive reaction.

### Statistical analysis

The course of infection was analyzed by a two-factor (treatment group and days) repeated-measures analysis of variance (ANOVA), and *post-hoc* analysis was completed using a Tukey–Kramer test. ELISA titers were analyzed by converting to an arithmetical scale and comparing the means using a two-way unpaired *t*-test.

### RESULTS

As shown in Figure 1, the oral administration of imiquimod did not alter the course of infection as assessed by shedding of viable *C. trachomatis*, MoPn. However, we did observe an effect of administration of the drug in that mice in the imiquimod-treated group consistently appeared to be somewhat morbid as evidenced by a lack of grooming, a loss of luster of the fur, and more agitation on handling. Similar changes were not observed in the mice that were treated with saline only. In order to determine whether the administration of imiquimod enhanced the Th1 response or otherwise affected the Th1 to Th2 polarization, IgG subclass antibody responses were assessed using ELISA. Antibody levels on day 0 were consistently below the detectable range at the lowest dilution assessed (1:10; data not shown). As shown in Figure 2, both groups displayed an IgG antibody subclass profile indicative of a vigorous Th1 response (IgG2a predominating over IgG1)[4]. No change in the overall antibody response of any IgG subclass was affected by imiquimod treatment. This indicated that the drug did not induce a shift in Th1 to Th2 immune response polarization as measured by this method, nor was an enhanced Th1 response evident.

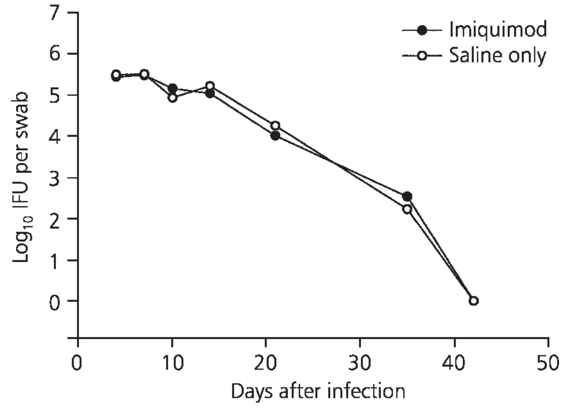

**Figure 1** Shedding of viable *C. trachomatis*, mouse pneumonitis strain, following intravaginal inoculation in mice that were administered imiquimod ($n = 47$, filled circles) or saline ($n = 49$, open circles) by gavage. Symbols represent the mean inclusion-forming units (IFU) isolated from cervical–vaginal swabs at each time point. No differences were observed when the courses of infection for the two groups were compared by two-factor (treatment group and days) repeated-measures analysis of variance (ANOVA) with *post-hoc* analysis using a Tukey–Kramer test

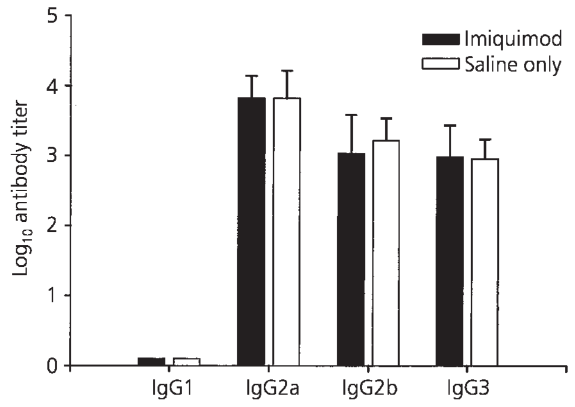

**Figure 2** Immmunoglobulin G (IgG) subclass antibody response at day 56 after infection following *C. trachomatis* (mouse pneumonitis strain) infection in mice that were administered imiquimod ($n = 7$, filled bars) or saline ($n = 6$, open bars). Each bar represents the mean $log_{10}$ antibody titer for each group. No differences were observed in any IgG subclass antibody response when compared using a two-way unpaired *t*-test

One explanation for the lack of effect of imiquimod in our first experiment could be that we used a systemic route for administration of the drug, whereas the infection in its earliest stages is localized to the genital epithelium. Therefore we repeated the experiment using a local, topically applied preparation of imiquimod (Aldara, 5% imiquimod cream). The experiment was of similar design to the previous one except that 10 μl of 5% imiquimod cream were applied intravaginally as described in the Materials and Methods section. As in the first experiment, blood was collected on the day of infection and again at 56 days after infection.

Figure 3 shows that vaginal application of 5% imiquimod cream did not alter the course of infection. Furthermore, as shown in Figure 4, administration of imiquimod intravaginally did not induce a shift in the Th1 to Th2 immune response as indicated by a change in the IgG antibody subclass responses. As observed in the first experiment, background levels of antibody in all IgG subclasses remained below detectable levels (data not shown). Interestingly, in this experiment we consistently detected a low IgG1 response in both groups of mice, which may indicate a slight shift toward a type-2 response phenotype. This response was also observed in the vehicle-only treatment group, indicating that the effect may be due to altered antigen penetration and presentation at the site of infection due to the vehicle. However, this is not at all conclusive, because the IgG1 responses were not compared in a protocol where oral and vaginal administration of imiquimod were compared in the same experiment.

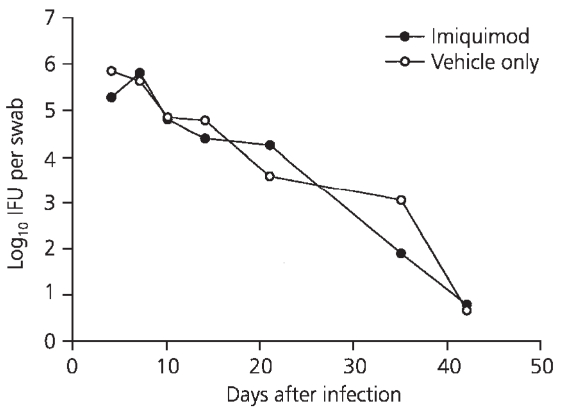

**Figure 3** Shedding of viable *C. trachomatis*, mouse pneumonitis strain, following intravaginal inoculation in mice that were administered imiquimod ($n$ = 10, filled circles) or vehicle only ($n$ = 10, open circles) intravaginally. Symbols represent the mean IFU isolated from cervical–vaginal swabs at each time point. No differences were observed when the courses of infection for the two groups were compared by two-factor (treatment group and days) repeated-measures ANOVA with *post-hoc* analysis using a Tukey–Kramer test

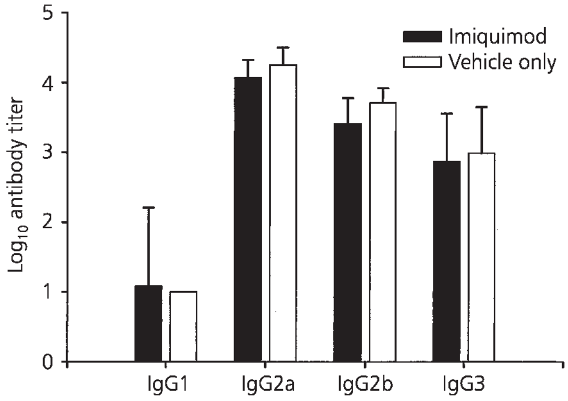

**Figure 4** IgG subclass antibody response at day 56 after infection following *C. trachomatis* (mouse pneumonitis strain) infection in mice that were administered imiquimod ($n$ = 5, filled bars) or vehicle only ($n$ = 5, open bars). Each bar represents the mean $\log_{10}$ antibody titer for each group. No differences were observed in any IgG subclass antibody response when compared using a two-way unpaired $t$-test

### DISCUSSION

Our results indicate that imiquimod affected neither resolution of chlamydial genital infection in this model nor reduced shedding of *C. trachomatis* (MoPn), whether administered locally at the site of infection or systemically via the oral route. This drug also does not induce an obvious shift in IgG antibody subclass responses that would by extension indicate either an enhanced Th1 response or a significant shift toward Th2 polarization in response to the infection in this model.

Although these results do not hold promise for imiquimod in therapy for chlamydial infection, we cannot rule out the possibility of its use to assess the immune response in certain other aspects of the infection. For example, in the context of this study, we did not assess pathological outcome subsequent to infection resolution. It is possible that although the drug did not alter the course of infection, chronic sequelae such as infertility[31] and hydrosalpinx[32,33] formation might be affected by host responses that were induced by the drug but not measured in our study. In addition, although neither a distinct shift from Th1 to Th2 polarization nor an enhancement of the natural Th1 response was observed, it is possible that any effect of the drug in this regard was masked due to the obvious potent Th1 response that develops naturally in response to this infection. This was evidenced by the high level of IgG2a production illustrated in Figures 2 and 4, and it has been reported elsewhere[34]. Although we chose the BALB/c strain of mice due to the fact that they are characterized as being moderately susceptible in this model, they do show many features of a Th1 response. Perhaps an effect would have been observed in a more susceptible strain, such as the C3H/HeN[18]. Thus alteration of a naturally potent Th1 response or overcoming the genetic composition of the host by chemical manipulation may prove difficult. A final explanation for the lack of effect of imiquimod in this model may relate to the actual mode of action of this drug. It has recently been shown that in mice imiquimod acts through the Toll-like-receptor-7 (TLR7)-MyD88-dependent signaling pathway[34]. Although these receptors are known to be expressed in certain leukocyte populations, their anatomic distribution within the mouse has not yet been described in detail. Thus if TLR7-MyD88 receptors are not expressed in sufficient quantities in the mouse in the genital epithelium or in tissues immediately adjacent to the latter, the lack of an adequate local target for the drug may explain its lack of effect in this model.

Kelly *et al.*[35] found that the route of exposure to viable *C. trachomatis* (MoPn) determined the type of response profile that was elicited. They found that subcutaneous administration of viable *C. trachomatis* (MoPn), yielded a predominant Th2 response, whereas administration by mucosal routes (oral, intranasal and vaginal) yielded a predominant Th1 polarization. Thus although an effect of imiquimod on infection and immune response was not observed, this drug might yet prove to have efficacy in altering immune response profiles subsequent to artificial immunization against chlamydial antigen by other routes. This may be particularly likely if it is found that TLR7-MyD88 expression densities vary between tissues as described above.

### ACKNOWLEDGEMENTS

The authors wish to thank Michael A. Yanez and Ira M. Sigar for their excellent technical assistance, and Linda S. Izzo and Angelo A. Izzo for helping with the gavaging of mice in the first experiment.

This research was sponsored in part by 3M Pharmaceuticals.

### REFERENCES

1. Dockrell DH, Kinghorn GR. Imiquimod and resiquimod as novel immunomodulators. *J Antimicrob Chemother* 2001;48:751–5
2. Gupta AK, Browne M, Bluhm R. Imiquimod: a review. *J Cutan Med Surg* 2002;6:554–60
3. Hengge U R, Benninghoff B, Ruzicka T, *et al.* Topical immunomodulators – progress towards treating inflammation, infection and cancer. *Lancet Infect Dis* 2001;1:189–98
4. Snapper CM, Paul WE. Interferon-gamma and B-cell stimulatory factor-1 reciprocally regulate Ig isotype production. *Science* 1987;236:944–7
5. Hengge UR, Esser S, Schultewolter T, *et al.* Self-administered topical 5% imiquimod for the treatment of common warts and molluscum contagiosum. *Br J Dermatol* 2000;143:1026–31
6. Hengge UR, Goos M, Arndt R. Topical treatment of warts and mollusca with imiquimod. *Ann Intern Med* 2000;132:95
7. Bernstein DI, Harrison CJ. Effects of the immunomodulating agent R837 on acute and latent herpes simplex virus type 2 infections. *Antimicrob Agents Chemother* 1989;33:1511–15
8. Gilbert J, Drehs MM, Weinberg JM. Topical imiquimod for acyclovir-unresponsive herpes simplex virus 2 infection. *Arch Dermatol* 2001; 137:1015–17
9. Harrison CJ, Stanberry LR, Bernstein DI. Effects of cytokines and R-837, a cytokine inducer, on UV-irradiation-augmented recurrent genital herpes in guinea pigs. *Antiviral Res* 1991;15: 315–22

10. Harrison CJ, Jenski L, Voychehovski T, *et al.* Modification of immunological responses and clinical disease during topical R-837 treatment of genital HSV-2 infection. *Antiviral Res* 1988; 10:209–23
11. Harrison CJ, Miller RL, Bernstein DI. Post-therapy suppression of genital herpes simplex virus (HSV) recurrences and enhancement of HSV-specific T-cell memory by imiquimod in guinea pigs. *Antimicrob Agents Chemother* 1994;38:2059–64
12. Chen M, Griffith BP, Lucia HL, *et al.* Efficacy of S26308 against guinea pig cytomegalovirus infection. *Antimicrob Agents Chemother* 1988; 32:678–83
13. Buates S, Matlashewski G. Treatment of experimental leishmaniasis with the immunomodulators imiquimod and S-28463: efficacy and mode of action. *J Infect Dis* 1999;179:1485–94
14. Arevalo I, Ward B, Miller R, *et al.* Successful treatment of drug-resistant cutaneous leishmaniasis in humans by use of imiquimod, an immunomodulator. *Clin Infect Dis* 2001;33:1847–51
15. Sauder DN. Immunomodulatory and pharmacologic properties of imiquimod. *J Am Acad Dermatol* 2000;43:S6–11
16. Brunham RC. Human immunity to *Chlamydia*. In Stephens RS, ed. Chlamydia: *Intracellular Biology, Pathogenesis and Immunity*. Washington, DC: ASM Press, 1999:211–38
17. Beatty WL, Morrison RP, Byrne GI. Persistent Chlamydiae: from cell culture to a paradigm for chlamydial pathogenesis. *Microbiol Rev* 1994;58: 686–99
18. Rank RG. Models of immunity. In Stephens RJ, ed. Chlamydia: *Intracellular Biology, Pathogenesis and Immunity*. Washington, DC: ASM Press, 1999: 239–95
19. Rank RG, Soderberg LSF, Barron AL. Chronic chlamydial genital infection in congenitally athymic nude mice. *Infect Immun* 1985;48:847–9
20. Ramsey KH, Soderberg LSF, Rank RG. Resolution of chlamydial genital infection in B-cell-deficient mice and immunity to reinfection. *Infect Immun* 1988;56:1320–5
21. Ramsey KH, Rank RG. Resolution of chlamydial genital infection of mice with antigen-specific T-lymphocyte lines. *Infect Immun* 1991;59:925–31
22. Rank RG, Ramsey KH, Pack EA, *et al.* Effect of gamma-interferon on resolution of murine chlamydial genital infection. *Infect Immun* 1992; 60:4427–9
23. Igietseme JU, Ramsey KH, Magee DM, *et al.* Resolution of murine chlamydial genital infection by the adoptive transfer of a biovar-specific Th1 T-cell clone. *Reg Immunol* 1994;5:317–24
24. Kelly KA, Rank RG. Identification of homing receptors that mediate the recruitment of CD4 T-cells to the genital tract following intravaginal infection with *Chlamydia trachomatis*. *Infect Immun* 1997;65:5198–208
25. Su H, Messer R, Whitmire W, *et al.* Vaccination against chlamydial genital tract infection after immunization with dendritic cells pulsed *ex vivo* with nonviable Chlamydiae. *J Exp Med* 1998; 188:809–18
26. Cotter TW, Ramsey KH, Miranpuri GS, *et al.* Dissemination of *Chlamydia trachomatis* chronic genital tract infection in gamma interferon gene knockout mice. *Infect Immun* 1997;65:2145–52
27. Morrison SG, Su H, Caldwell HD, *et al.* Immunity to murine *Chlamydia trachomatis* genital tract reinfection involves B-cells and CD4(+) T-cells but not CD8(+) T-cells. *Infect Immun* 2000;68: 6979–87
28. Moore T, Ananaba GA, Bolier J, *et al.* Fc receptor regulation of protective immunity against *Chlamydia trachomatis*. *Immunology* 2002;105: 213–21
29. Cotter TW, Meng Q, Shen Z, *et al.* Protective efficacy of major outer membrane protein specific immunoglobulin A (IgA) and IgG monoclonal antibodies in a murine model of *Chlamydia trachomatis* genital tract infection. *Infect Immun* 1996;63:4704–14
30. Ramsey KH, Newhall WJ, Rank RG. Humoral immune response to chlamydial genital infection of mice with the agent of mouse pneumonitis. *Infect Immun* 1989;57:2441–6
31. De La Maza LM, Pal S, Khamesipour A, *et al.* Intravaginal inoculation of mice with the *Chlamydia trachomatis* mouse pneumonitis biovar results in infertility. *Infect Immun* 1994;62:2094–7
32. Ramsey KH, Sigar IM, Rana S, *et al.* Role for inducible nitric oxide synthase in protection from chronic *Chlamydia trachomatis* urogenital disease in mice and its regulation by oxygen-free radicals. *Infect Immun* 2001;69:7374–9
33. Ramsey KH, Miranpuri GS, Sigar IM, *et al.* *Chlamydia trachomatis* persistence in the female mouse genital tract: inducible nitric oxide synthase and infection outcome. *Infect Immun* 2001; 69:5131–7

34. Hemmi H, Kaisho T, Takeuchi O, *et al*. Small anti-viral compounds activate immune cells via the TLR7 MyD88-dependent signaling pathway. *Nat Immunol* 2002;3:196–200
35. Kelly KA, Robinson EA, Rank RG. Initial route of antigen administration alters the T-cell cytokine profile produced in response to the mouse pneumonitis biovar of *Chlamydia trachomatis* following genital infection. *Infect Immun* 1996; 64:4976–83

RECEIVED 07/08/02; ACCEPTED 04/11/03

## References

[PDF_00455] Arevalo I., Ward B., Miller R., Meng T. C., Najar E., Alvarez E., Matlashewski G., Llanos-Cuentas A. (2001). Successful treatment of drug-resistant cutaneous leishmaniasis in humans by use of imiquimod, an immunomodulator.. Clin Infect Dis.

[PDF_00466] Beatty W. L., Morrison R. P., Byrne G. I. (1994). Persistent chlamydiae: from cell culture to a paradigm for chlamydial pathogenesis.. Microbiol Rev.

[PDF_00417] Bernstein D. I., Harrison C. J. (1989). Effects of the immunomodulating agent R837 on acute and latent herpes simplex virus type 2 infections.. Antimicrob Agents Chemother.

[PDF_00451] Buates S., Matlashewski G. (1999). Treatment of experimental leishmaniasis with the immunomodulators imiquimod and S-28463: efficacy and mode of action.. J Infect Dis.

[PDF_00447] Chen M., Griffith B. P., Lucia H. L., Hsiung G. D. (1988). Efficacy of S26308 against guinea pig cytomegalovirus infection.. Antimicrob Agents Chemother.

[PDF_00515] Cotter T. W., Meng Q., Shen Z. L., Zhang Y. X., Su H., Caldwell H. D. (1995). Protective efficacy of major outer membrane protein-specific immunoglobulin A (IgA) and IgG monoclonal antibodies in a murine model of Chlamydia trachomatis genital tract infection.. Infect Immun.

[PDF_00502] Cotter T. W., Ramsey K. H., Miranpuri G. S., Poulsen C. E., Byrne G. I. (1997). Dissemination of Chlamydia trachomatis chronic genital tract infection in gamma interferon gene knockout mice.. Infect Immun.

[PDF_00398] Dockrell D. H., Kinghorn G. R. (2001). Imiquimod and resiquimod as novel immunomodulators.. J Antimicrob Chemother.

[PDF_00421] Gilbert J., Drehs M. M., Weinberg J. M. (2001). Topical imiquimod for acyclovir-unresponsive herpes simplex virus 2 infection.. Arch Dermatol.

[PDF_00401] Gupta Aditya K., Browne Melanie, Bluhm Robyn (2002). Imiquimod: a review.. J Cutan Med Surg.

[PDF_00437] Harrison C. J., Jenski L., Voychehovski T., Bernstein D. I. (1988). Modification of immunological responses and clinical disease during topical R-837 treatment of genital HSV-2 infection.. Antiviral Res.

[PDF_00442] Harrison C. J., Miller R. L., Bernstein D. I. (1994). Posttherapy suppression of genital herpes simplex virus (HSV) recurrences and enhancement of HSV-specific T-cell memory by imiquimod in guinea pigs.. Antimicrob Agents Chemother.

[PDF_00425] Harrison C. J., Stanberry L. R., Bernstein D. I. (1991). Effects of cytokines and R-837, a cytokine inducer, on UV-irradiation augmented recurrent genital herpes in guinea pigs.. Antiviral Res.

[PDF_00546] Hemmi Hiroaki, Kaisho Tsuneyasu, Takeuchi Osamu, Sato Shintaro, Sanjo Hideki, Hoshino Katsuaki, Horiuchi Takao, Tomizawa Hideyuki, Takeda Kiyoshi, Akira Shizuo (2002). Small anti-viral compounds activate immune cells via the TLR7 MyD88-dependent signaling pathway.. Nat Immunol.

[PDF_00403] Hengge U. R., Benninghoff B., Ruzicka T., Goos M. (2001). Topical immunomodulators--progress towards treating inflammation, infection, and cancer.. Lancet Infect Dis.

[PDF_00410] Hengge U. R., Esser S., Schultewolter T., Behrendt C., Meyer T., Stockfleth E., Goos M. (2000). Self-administered topical 5% imiquimod for the treatment of common warts and molluscum contagiosum.. Br J Dermatol.

[PDF_00414] Hengge U. R., Goos M., Arndt R. (2000). Topical treatment of warts and mollusca with imiquimod.. Ann Intern Med.

[PDF_00488] Igietseme J. U., Ramsey K. H., Magee D. M., Williams D. M., Kincy T. J., Rank R. G. (1993). Resolution of murine chlamydial genital infection by the adoptive transfer of a biovar-specific, Th1 lymphocyte clone.. Reg Immunol.

[PDF_00492] Kelly K. A., Rank R. G. (1997). Identification of homing receptors that mediate the recruitment of CD4 T cells to the genital tract following intravaginal infection with Chlamydia trachomatis.. Infect Immun.

[PDF_00550] Kelly K. A., Robinson E. A., Rank R. G. (1996). Initial route of antigen administration alters the T-cell cytokine profile produced in response to the mouse pneumonitis biovar of Chlamydia trachomatis following genital infection.. Infect Immun.

[PDF_00511] Moore Terri, Ananaba Godwin A., Bolier Jacqueline, Bowers Samera, Belay Tesfaye, Eko Francis O., Igietseme Joseph U. (2002). Fc receptor regulation of protective immunity against Chlamydia trachomatis.. Immunology.

[PDF_00506] Morrison S. G., Su H., Caldwell H. D., Morrison R. P. (2000). Immunity to murine Chlamydia trachomatis genital tract reinfection involves B cells and CD4(+) T cells but not CD8(+) T cells.. Infect Immun.

[PDF_00534] Ramsey K. H., Miranpuri G. S., Sigar I. M., Ouellette S., Byrne G. I. (2001). Chlamydia trachomatis persistence in the female mouse genital tract: inducible nitric oxide synthase and infection outcome.. Infect Immun.

[PDF_00521] Ramsey K. H., Newhall W. J., Rank R. G. (1989). Humoral immune response to chlamydial genital infection of mice with the agent of mouse pneumonitis.. Infect Immun.

[PDF_00481] Ramsey K. H., Rank R. G. (1991). Resolution of chlamydial genital infection with antigen-specific T-lymphocyte lines.. Infect Immun.

[PDF_00529] Ramsey K. H., Sigar I. M., Rana S. V., Gupta J., Holland S. M., Byrne G. I. (2001). Role for inducible nitric oxide synthase in protection from chronic Chlamydia trachomatis urogenital disease in mice and its regulation by oxygen free radicals.. Infect Immun.

[PDF_00477] Ramsey K. H., Soderberg L. S., Rank R. G. (1988). Resolution of chlamydial genital infection in B-cell-deficient mice and immunity to reinfection.. Infect Immun.

[PDF_00484] Rank R. G., Ramsey K. H., Pack E. A., Williams D. M. (1992). Effect of gamma interferon on resolution of murine chlamydial genital infection.. Infect Immun.

[PDF_00474] Rank R. G., Soderberg L. S., Barron A. L. (1985). Chronic chlamydial genital infection in congenitally athymic nude mice.. Infect Immun.

[PDF_00459] Sauder D. N. (2000). Immunomodulatory and pharmacologic properties of imiquimod.. J Am Acad Dermatol.

[PDF_00407] Snapper C. M., Paul W. E. (1987). Interferon-gamma and B cell stimulatory factor-1 reciprocally regulate Ig isotype production.. Science.

[PDF_00497] Su H., Messer R., Whitmire W., Fischer E., Portis J. C., Caldwell H. D. (1998). Vaccination against chlamydial genital tract infection after immunization with dendritic cells pulsed ex vivo with nonviable Chlamydiae.. J Exp Med.

[PDF_00525] de la Maza L. M., Pal S., Khamesipour A., Peterson E. M. (1994). Intravaginal inoculation of mice with the Chlamydia trachomatis mouse pneumonitis biovar results in infertility.. Infect Immun.

